# Nanoparticle‐microglial interaction in the ischemic brain is modulated by injury duration and treatment

**DOI:** 10.1002/btm2.10175

**Published:** 2020-08-15

**Authors:** Andrea Joseph, Rick Liao, Mengying Zhang, Hawley Helmbrecht, Michael McKenna, Jeremy R. Filteau, Elizabeth Nance

**Affiliations:** ^1^ Department of Chemical Engineering University of Washington Seattle Washington USA; ^2^ Molecular Engineering and Sciences Institute University of Washington Seattle Washington USA; ^3^ Department of Radiology University of Washington Seattle Washington USA; ^4^ eScience Institute University of Washington Seattle Washington USA

**Keywords:** dendrimer, diffusion, drug delivery, hypoxia‐ischemia, organotypic slices, polymeric nanoparticles

## Abstract

Cerebral ischemia is a major cause of death in both neonates and adults, and currently has no cure. Nanotechnology represents one promising area of therapeutic development for cerebral ischemia due to the ability of nanoparticles to overcome biological barriers in the brain. ex vivo injury models have emerged as a high‐throughput alternative that can recapitulate disease processes and enable nanoscale probing of the brain microenvironment. In this study, we used oxygen–glucose deprivation (OGD) to model ischemic injury and studied nanoparticle interaction with microglia, resident immune cells in the brain that are of increasing interest for therapeutic delivery. By measuring cell death and glutathione production, we evaluated the effect of OGD exposure time and treatment with azithromycin (AZ) on slice health. We found a robust injury response with 0.5 hr of OGD exposure and effective treatment after immediate application of AZ. We observed an OGD‐induced shift in microglial morphology toward increased heterogeneity and circularity, and a decrease in microglial number, which was reversed after treatment. OGD enhanced diffusion of polystyrene‐poly(ethylene glycol) (PS‐PEG) nanoparticles, improving transport and ability to reach target cells. While microglial uptake of dendrimers or quantum dots (QDs) was not enhanced after injury, internalization of PS‐PEG was significantly increased. For PS‐PEG, AZ treatment restored microglial uptake to normal control levels. Our results suggest that different nanoparticle platforms should be carefully screened before application and upon doing so; disease‐mediated changes in the brain microenvironment can be leveraged by nanoscale drug delivery devices for enhanced cell interaction.

## Graphical abstract

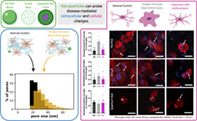

## 1 INTRODUCTION

Cerebral ischemia is a major cause of death in both neonates and adults.^1^, ^2^ Unfortunately, treatments for both neonatal hypoxia‐ischemia (HI) and adult stroke provide only modest benefits in mortality and morbidity.^3^, ^4^ Investigation of effective treatments for HI continues to be a critical area of research. Microglia, the resident immune cells of the brain are of more recent and special interest for therapeutic targeting in HI.^5^ Microglia become activated after ischemic injury, exhibit increased phagocytic behavior, and contribute to neuroinflammatory and reactive oxygen species (ROS) stress that may exacerbate damage in the brain.^6^ Thus, an opportunity exists to provide neuroprotection after ischemic injury by designing therapeutics to target and modulate microglial behavior.

A promising strategy for microglial‐targeted therapeutic development is the delivery of drugs via nano‐sized carriers. Although nanoparticle platforms vary widely in composition, shape, and other physical characteristics, several distinct nanoparticle types have shown an ability to overcome biological barriers to drug delivery in the brain. For example, polymeric nanoparticles (size < 200 nm) with a dense poly(ethylene glycol) (PEG) coating can rapidly penetrate within small pores in the brain extracellular space (ECS) that restrict the diffusion and broad distribution of most therapeutics.^7^, ^8^ Adequate intracellular trafficking of therapeutics also presents a major drug delivery challenge, but nanoparticles can leverage existing endocytosis pathways in microglial cells for internalization. Quantum dots (QDs) and poly(amidoamine) (PAMAM) dendrimer nanoparticles, among others, have shown accumulation within activated microglia.^9^, ^10^, ^11^, ^12^, ^13^ While nanoparticles can facilitate and enhance drug transport in the brain, these effects are dependent on both nanoparticle characteristics and disease state,^14^ requiring further screening and study.

We investigate the role of injury, treatment, and nanoparticle type in driving nanoparticle‐microglial interactions in ischemic conditions. We use ex vivo organotypic whole hemisphere (OWH) brain slices, which have emerged as a high‐throughput platform for modeling disease processes and screening therapeutic platforms, including nanoparticles.^13^, ^15^, ^16^ The ability to obtain multiple OWH slices from a single brain reduces biological variation and enables detailed investigation of disease environments or therapeutic efficacy. OWH slices also preserve functional relationships between neighboring cells and maintain 3D‐cytoarchitecture.^15^ Importantly, oxygen–glucose deprivation (OGD) has been widely used to model ischemic injury in organotypic slices.^17^ OGD brain slice models retain in vivo pathological processes including extracellular glutamate release, neuronal damage, and production of cytokines and oxidative stress markers.^18^, ^19^, ^20^ Thus, OGD exposed OWH slices are a powerful tool for evaluation of nanoparticle–cell interactions in ischemic injury.

To establish the degree of injury and microglial response following OGD exposure, we evaluate cytotoxicity and oxidative stress in healthy, OGD exposed, and azithromycin (AZ)‐treated OWH brain slices. AZ is an FDA‐approved therapy that can suppress both acute and chronic pathologic microglial activation in response to ischemic stroke injury.^21^, ^22^ Because microglial behavior correlates with disease severity,^23^, ^24^ AZ modulation of microglia provides one avenue to study microglia‐nanoparticle interaction in response to treatment.^25^ We use a Python‐based image analysis technique to quantify the degree of microglial morphological heterogeneity following injury and treatment. We next investigate how injury alters the ability of nanoparticles to diffuse within the brain, an important factor for maximal distribution to reach target microglial cells. Last, we use flow cytometry and immunofluorescent imaging to quantify nanoparticle uptake in microglia based on injury and treatment. We compare three distinct nanoparticle platforms, polystyrene (PS)‐PEG, PAMAM dendrimers, and cadmium selenide/cadmium sulfide (CdSe/CdS) core/shell QDs, to determine the influence of nanoparticle physical characteristics on microglial uptake. In using OWH slices to probe nanoparticle‐microglial interaction after disease and treatment, our study informs the design of nanoparticles to leverage the brain microenvironment and target microglial cells for enhanced therapeutic outcome in ischemic conditions.

## 2 MATERIALS AND METHODS

### 2.1 Animal experiments and ethics statement

This study was performed in accordance with the guide for the care and use of laboratory animals of the National Institutes of Health (NIH). All animals were handled according to an approved Institutional Animal Care and Use Committee (IACUC) protocol (#4383‐02) of the University of Washington (UW), Seattle, WA. The UW has an approved Animal Welfare Assurance (#A3464‐01) on file with the NIH Office of Laboratory Animal Welfare, is registered with the United States Department of Agriculture (certificate #91‐R‐0001), and is accredited by AAALAC International. Animal housing conditions are provided in Supplemental Information.

### 2.2 Slice culturing, OGD, and treatment

OWH brain slice culturing techniques were prepared adapted from previously published methodology.^13^ Slice preparation and culturing medias are provided in Supplemental Information. For glutathione and flow cytometry measurements, three brain slices were plated per membrane insert. For all other experiments, one brain slice was plated per membrane insert. After slices rested overnight in the incubator, culture media was removed, and fresh media was added, followed by two more days of rest. Samples underwent OGD after 3 days in vitro (DIV), except for flow cytometry studies, which underwent 2 DIV. The end of the OGD incubation period was defined as time t = 0 hr. For the normal control (NC) condition, slices proceeded directly to t = 0 hr without OGD media exchange. For a subset of groups, 100 μl 1 x PBS containing 0.1 mg superoxide dismutase (SOD, Cu/Zn SOD1 from bovine erythrocytes, Sigma) or 0.75 μg (150 mg/kg brain tissue) AZ (Zithromax) per slice was added directly to the 3 and 0.5 hr OGD slices, respectively. Six‐well plates were returned to the CO_2_ incubator until further processing.

### 2.3 Cell health and image analysis

At t = 24 hr for all sample conditions, slices were stained with 5 μg/ml propidium iodide (PI), fixed, and stained with 4′,6‐diamidino‐2‐phenylindole (DAPI, Invitrogen, 1:10,000), goat anti‐Iba1 antibody (Wako 019–19,741, 1:200) and anti‐goat AlexaFluor 488 (Life Technologies A11034, 1:500) for microglia. Glutathione (GSH) assay and staining protocols are provided in Supplemental Information. Slices were imaged using a Nikon A1R with a ×40 objective. For every slice, five images were acquired from each brain region of interest (cortex and thalamus). Image acquisition settings were consistent for all images. For each image, DAPI+ cells (total cells) and PI+ cells (dead cells) were counted manually in ImageJ (NIH) after applying an Otsu threshold and fluorescent cutoff to aid in visualization. The PI+/DAPI+ cell ratio was expressed as the percentage of dead cells in an individual image. Image acquisition and analysis was performed in a blinded manner. Iba1 images were analyzed using an adaptation of Visually Aided Morpho‐Phenotyping Image Recognition (VAMPIRE).^26^

### 2.4 Nanoparticle preparation and characterization

Forty‐nanometer dark red fluorescent carboxylate (COOH)‐modified PS latex nanoparticles (PS‐COOH) (Thermo Fisher Scientific) were covalently modified with methoxy (MeO)‐PEG‐amine (NH_2_) (5 kDa MW, Creative PEG Works) by a carboxyl amine reaction.^27^ Generation‐4 hydroxyl modified PAMAM dendrimers labeled with Cy5 (D‐Cy5) were provided by Dr. Rangaramanujam Kannan and Dr. Anjali Sharma at the Johns Hopkins Center for Nanomedicine.^28^, ^29^ These conjugates are stable at physiological conditions and have been validated for ex vivo application at the concentration used in our study (1 ng/ul).^9^ CdSe/CdS core‐shell QDs with PEG‐methoxy functionality were provided by Dr Vince Holmberg in the Department of Chemical Engineering at the UW, which were proven stable at physiological conditions for ex vivo application.^13^ Nanoparticle characterization, multiple particle tracking (MPT) to measure nanoparticle diffusion, trajectory analysis^30^ and application of the Amsden obstruction‐scaling model to calculate effective pore sizes, including all assumptions,^31^, ^32^, ^33^, ^34^ are detailed in Supplemental Information.

### 2.5 Nanoparticle colocalization in microglia and neurons

To probe nanoparticle interactions with microglia, at t = 1 hr PS‐PEG, D‐Cy5, and QDs (10 μl of 1 ng/μl) were topically pipetted on NC, 0.5 h OGD, and 0.5 h OGD + AZ OWH slices. The slices were then live‐incubated for 4 hr to allow the nanoparticles to diffuse through the brain tissue and interact with microglia. Slices were fixed and imaged or processed for flow cytometry, as described in Supplemental Information.

### 2.6 VAMPIRE for microglial morphometric analysis

All confocal microscopy images were converted from the .nd2 to .tiff file format. Using Python, all images were separated by RGB channel and labeled with the appropriate cell stain: DAPI for the blue channel and Iba1 for the green channel.^35^, ^36^, ^37^ Every image was then split into four quadrants using Image_slicer. Scikit‐learn split all images in an 80:20 test‐to‐train ratio,^38^ assuring at least two images for each slice of the three experimental conditions: NC, 0.5 h OGD, and 0.5 h OGD + AZ. Cells from each image were segmented using Cell Profiler and the Cell Profiler pipeline associated with the VAMPIRE package.^39^ A model of shape modes was built from all training images using the VAMPIRE package and associated protocol (https://github.com/kukionfr/VAMPIRE_open),^26^ and then applied to all images. The shape mode frequencies of individual slices were averaged for resulting distribution plots. Equation (1) was used to calculate the difference in sample shape mode frequency from NC shape mode frequency:

(1) $$\text{Absolute difference}=\left|x_{n}-x_{\mathrm{NC},n}\right|,$$

where *n* is the shape mode (1 through 5), *x*_*n*_ is the sample frequency for shape mode *n*, and *x*_NC,*n*_ is the NC frequency for shape mode *n*. Circularity was calculated with Equation (2):

(2) $$\text{Circularity}=\frac{4\mathrm{\pi A}}{P^{2}},$$

where *A* is the area and *P* is the perimeter for each microglia.

### 2.7 Statistical analysis

All statistical analyzes were carried out in GraphPad Prism (GraphPad Software Inc, Version 8.4.0). The D'Agostino‐Pearson omnibus K2 test was used to test for normality. In all instances, we were able to reject the null hypothesis that the data were sampled from a normally distributed population with high confidence. The Mann–Whitney test was thus used to test for significance. For GSH concentrations, microglia NC deviation, and microglia circularity data, unpaired *t* test with Welch's correction was used to test for significance. We reported statistical significance for *p*‐value<.05 (*).

## 3 RESULTS

### 3.1 OGD time‐dependent severity

**FIGURE 1:**
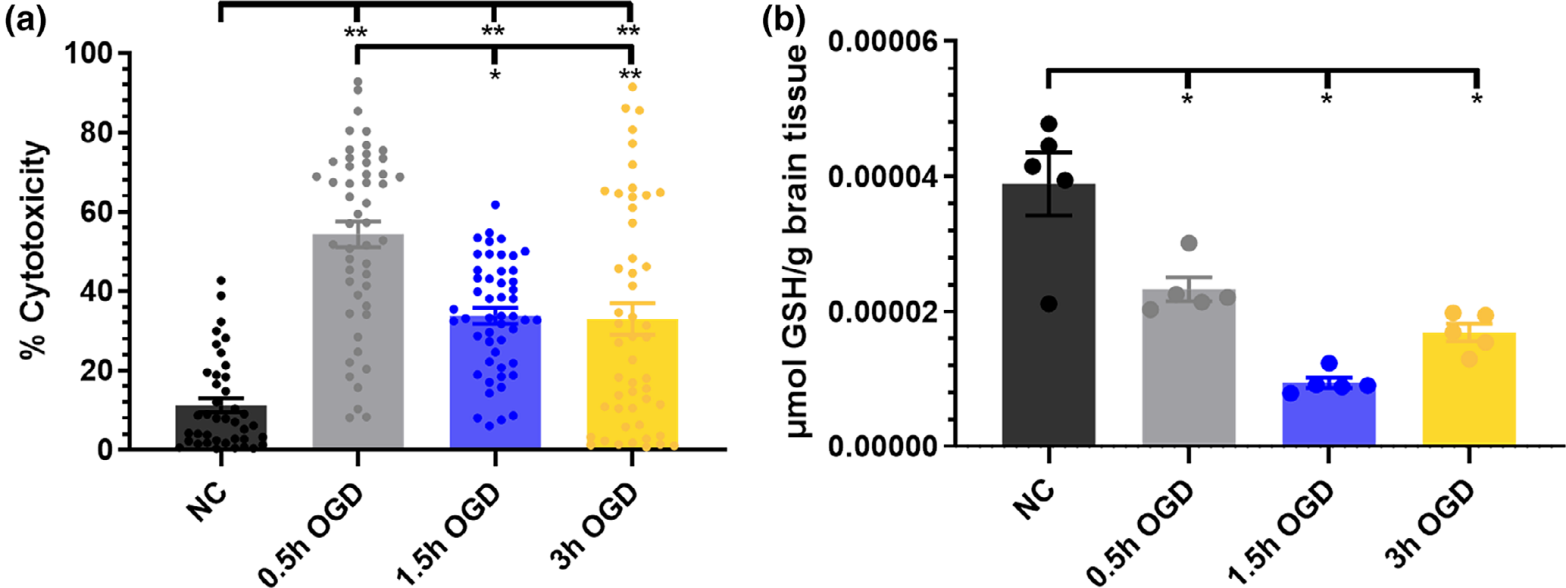
OGD exposure‐dependent effect on cell death and oxidative stress environment. (a) Percent cytotoxicity as determined by PI+/DAPI+ cell ratio (n = 40–50 per condition) and (b) GSH concentration (n = 5) for NC, 0.5, 1.5, and 3 hr OGD exposed brain slices. DAPI, diamidino‐2‐phenylindole; GSH, glutathione; NC, normal control; OGD, oxygen–glucose deprivation; PI, propidium iodide

HI duration can drastically change disease outcomes.^40^ Therefore, we first investigated the impact of OGD time on cell viability and the oxidative stress environment. Compared to NC slice cytotoxicity of 11.2%, 0.5, 1.5, and 3 hr OGD exposure times resulted in significant increases in cytotoxicity of 54.3, 33.8, and 32.9% respectively (Figure 1a). The 0.5 hr OGD cytotoxicity was also significantly higher than that of 1.5 or 3 hr OGD. 0.5, 1.5, and 3 hr OGD exposure times also yielded significantly decreased GSH concentrations of 1.7‐fold, 4.1‐fold, and 2.3‐fold reductions, respectively (Figure 1b).

### 3.2 Therapeutic effect of SOD and AZ on OGD‐induced injury

**FIGURE 2:**
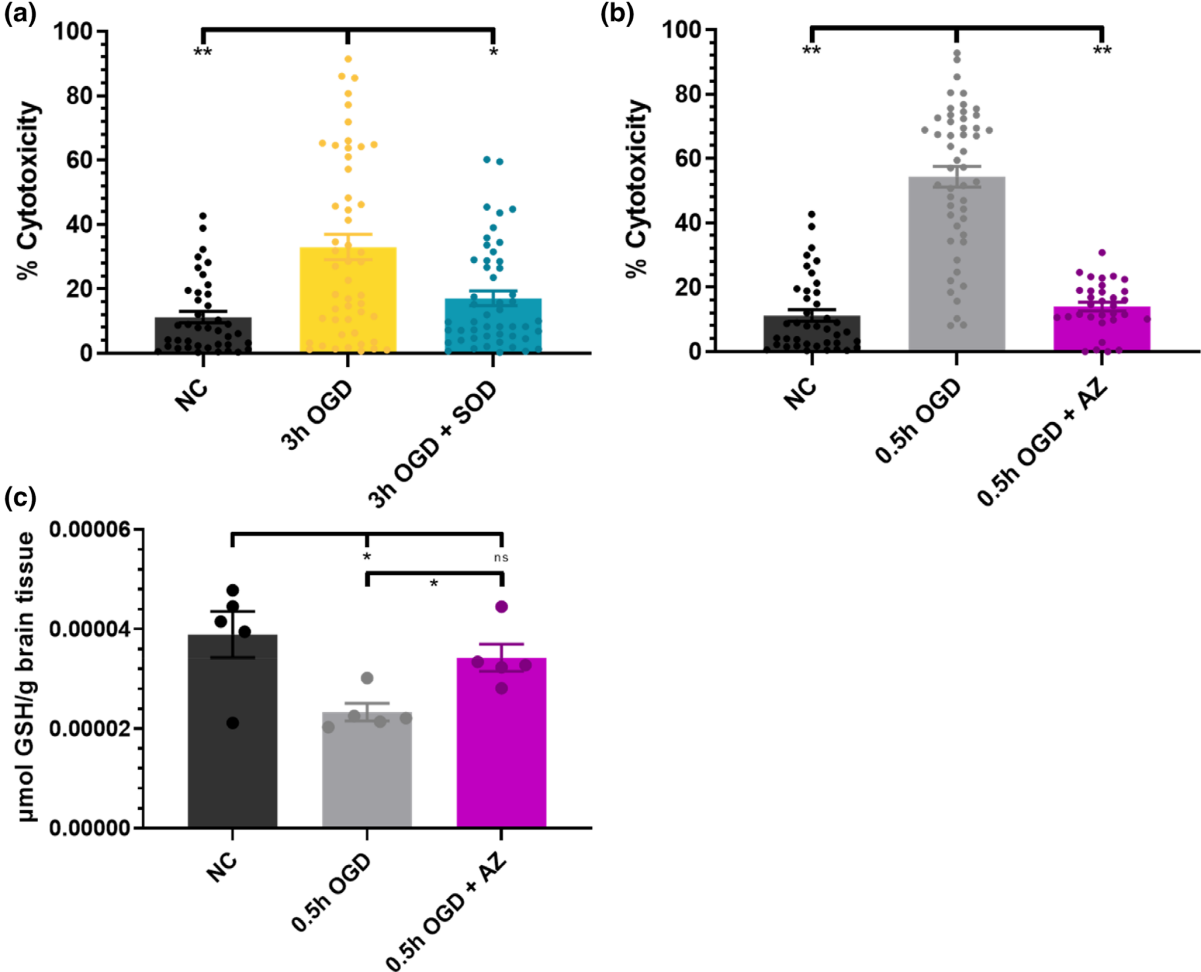
Therapeutic effects on OGD exposure‐induced injury. Percent cytotoxicity of (a) NC, 3 hr OGD, and 3 hr OGD + SOD (n = 40–50 per condition), (b) NC, 0.5 hr OGD, and 0.5 hr OGD + AZ (n = 33–50 per condition) measured by PI‐positive/DAPI‐positive cell ratio and (c) GSH concentrations of NC, 0.5 hr OGD, and 0.5 hr OGD + AZ (n = 5). NC, 0.5 hr OGD, and 3 hr OGD conditions are the same as presented in Figure 1. AZ, azithromycin; DAPI, diamidino‐2‐phenylindole; GSH, glutathione; NC, normal control; OGD, oxygen–glucose deprivation; PI, propidium iodide; SOD, superoxide dismutase

Prior to the investigation of AZ effects on nanoparticle interaction with microglia, we evaluated the effect of AZ and SOD on OGD‐induced cytotoxicity to confirm therapeutic effects seen in literature. We have previously shown SOD can attenuate excitotoxic damage in OWH brain slices.^15^ SOD addition to 3 hr OGD exposed slices significantly reduced cytotoxicity of 17.1% (*p* = .036) (Figure 2a). Having observed the greatest cytotoxicity induced by 0.5 hr OGD compared to NC, we proceeded to investigate the cytotoxicity effect of AZ on 0.5 hr OGD exposed slices. AZ treatment significantly reduced cytotoxicity to 14.0% (*p* < .001) (Figure 2b). AZ treatment also significantly increased GSH concentration 1.5‐fold compared to that of 0.5 hr OGD (*p* = .013) (Figure 2c).

### 3.3 OGD and AZ effects on microglial shape as determined by VAMPIRE

**FIGURE 3:**
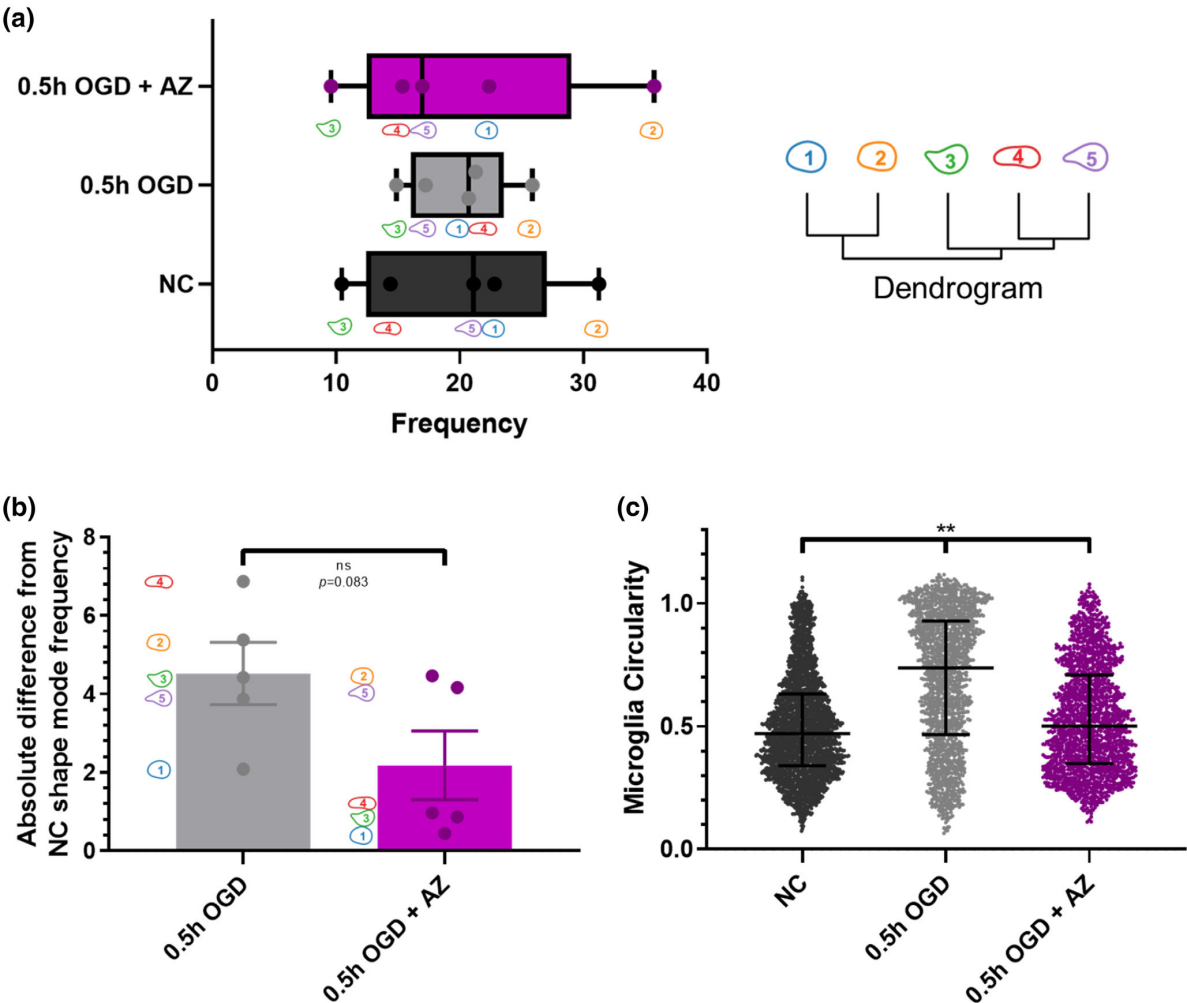
AZ reverses microglial heterogeneity and circularity after 0.5 h OGD. (a) Frequency of five shape modes of microglia for NC, 0.5 hr OGD, and 0.5 hr OGD + AZ groups as generated from the VAMPIRE package, (b) Absolute difference in shape mode frequencies of 0.5 hr OGD and 0.5 hr OGD + AZ from NC shape mode frequencies and (c) Microglial circularity of NC (n = 2,954 microglia from 4 slices), 0.5 hr OGD (n = 1,539 microglia from five slices), and 0.5 hr OGD + AZ (n = 1,612 microglia from three slices). AZ, azithromycin; NC, normal control; OGD, oxygen–glucose deprivation; VAMPIRE, Visually Aided Morpho‐Phenotyping Image Recognition

To better understand microglial response to OGD injury, we characterized microglial morphological heterogeneity across disease states. Microglial morphology and heterogeneity are dependent on the disease environment and are one indicator of microglial phenotype and function.^41^, ^42^ Relevant to this study, nanoparticle uptake has been correlated to microglial activation state,^9^, ^43^ with similar findings in liver‐derived macrophages, where phenotype determined nanoparticle uptake.^44^ Confocal images of microglia from each group were used to train a model via VAMPIRE,^26^ which resulting in five distinct microglial shape modes and subsequent classification of each microglial cell into its closest matching shape mode. The frequency of the five shape modes for each group is the percentage of microglia that exhibited the given shape mode (Figure 3a). 0.5 hr OGD with AZ treatment resulted in a distribution of shape modes similar to that of NC microglia: shape modes 3, 4, 5, 1, and 2 in order of increasing frequency. Overall, 0.5 hr OGD showed a reduced spread in shape mode frequencies, indicating an increase in microglial shape heterogeneity since all five shape modes were more equally represented. The absolute difference of shape mode frequency from NC shape mode frequency was 4.5 and 2.2% for 0.5 and 0.5 h OGD + AZ, respectively, although the difference between the two groups was not significant (*p* = .083) (Figure 3b). Circularity was calculated directly from individual microglia. Upon 0.5 hr OGD exposure, microglia exhibited a significantly increased circularity of 0.69 compared to NC circularity of 0.50 (*p* < .001) (Figure 3c). AZ treatment reversed the extent of microglial circularity to 0.53, significantly lower than that of 0.5 hr OGD (*p* < .001). NC and OGD + AZ circularity were also significantly different (*p* < .001). Furthermore, circularity did not correlate to shape mode, with a greater circularity for the 0.5 hr OGD condition for each of the five shape modes (Supplemental Table 1).

### 3.4 OGD enhances nanoparticle diffusion through the brain ECS

**FIGURE 4:**
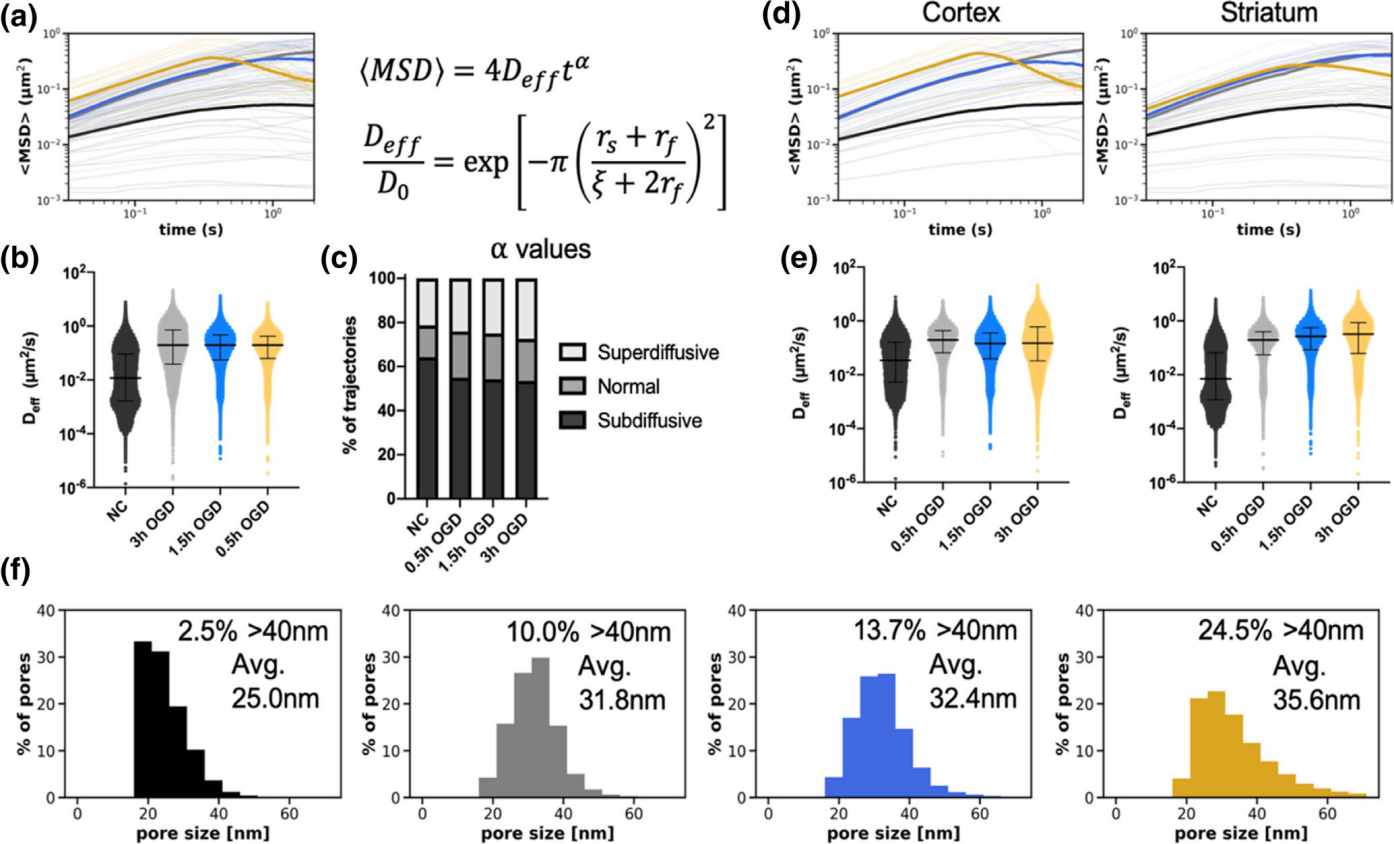
Characterization of nanoparticle diffusive behavior after OGD. (a) Geometrically ensemble‐averaged MSD versus lag time for NC (black), 0.5 hr (gray), 1.5 hr (blue), and 3 hr OGD (gold) conditions. Faint lines represent averages of each video (30 total per group) and the bolded line represents the mean of all videos, (b) *D*_eff_ values extracted from each trajectory from all videos (n = 10) in all slices (n = 3) per group (1 dot = 1 trajectory), (c) The anomalous exponent α was calculated by fitting trajectory MSDs to the anomalous diffusion equation. α was used to classify trajectories as either superdiffusive, normal, or subdiffusive, (d) <MSD> versus lag time split by brain region (left: cortex, right: striatum), (e) *D*_eff_ values split by brain region: (left = cortex, right = striatum), and (f) Effective ECS pore size distributions in NC and 0.5, 1.5, and 3 hr OGD slices from left to right. % of pores >40 nm and average pore size are indicated by text. Data for b and e are reported as median ± interquartile range. ECS, extracellular space; MSD, mean squared displacements; NC, normal control; OGD, oxygen–glucose deprivation

Nanotherapeutics must be able to reach target cells from the point of entry in the brain. To probe the effect of OGD exposure on a nanoparticle's ability to move, we performed MPT with 40 nm PS‐PEG nanoparticles in ex vivo slices after OGD. Particle characteristics are in Supplemental Table 2. Mean squared displacements (MSD) were calculated for each trajectory, and the geometrically ensemble‐averaged MSD (<MSD>) for each experimental group was generated as a function of lag time (Figure 4a). Regardless of OGD duration, nanoparticles showed greater displacement, indicating an elevated ability to diffuse compared to the NC group. Extraction of *D*_eff_ values using the Einstein‐Smoluchowski Equation revealed a 16.7‐, 16.5‐, and 16.6‐fold increase in nanoparticle diffusive ability in the 0.5, 1.5, and 3 hr OGD groups, respectively, compared to that of the NC group (Figure 4b). The <MSD> curves were then fit using the anomalous diffusion equation to obtain values of the anomalous diffusion exponent, α. Trajectories were classified as either superdiffusive (α > 1.25), normal (0.75 ≤ α ≤ 1.25), or subdiffusive (α < 0.75) (Figure 4c). OGD increased superdiffusive and decreased subdiffusive transport for all exposure times. The proportion of superdiffusive trajectories increased by 6.1% while subdiffusion decreased by 10.8% between the NC and 3 hr OGD groups. Changes in diffusion were region‐dependent (Figure 4d); the 0.5, 1.5, and 3 hr OGD exposure increased diffusion by 5.8‐, 4.3‐, and 4.5‐fold, respectively, in the cortex compared to 27.8‐, 38.0‐, and 45.8‐fold, respectively, in the striatum (Figure 4e). The regional and combined median *D*_eff_ and trajectory counts are presented in Table 1.

**TABLE 1:** *D*_eff_ in rat brain tissue were extracted at τ = 0.303 s from nanoparticle trajectories in the cortex (n = 5 videos per slice) and striatum (n = 5 videos per slice) in n = 3 slices per group

Treatment	Trajectories in cortex	*D*_eff_ in cortex (μm^2^/s)	Trajectories in striatum	*D*_eff_ in striatum (μm^2^/s)	Median *D*_eff_ (μm^2^/s)
NC	24,382	0.0338	64,840	0.00707	0.0118
0.5 hr OGD	65,305	0.197	42,378	0.196	0.197
1.5 hr OGD	43,520	0.146	38,787	0.269	0.195
3 hr OGD	103,415	0.151	51,285	0.324	0.195

Abbreviations: NC, normal control; OGD, oxygen–glucose deprivation.

We also anticipated that OGD exposure would alter the ECS through which nanoparticles can move. To estimate the distribution of effective ECS pores, we fit the Amsden obstruction‐scaling model for entangled and cross‐linked hydrogels^31^ to the *D*_eff_ data in Figure 4b. As exposure to OGD increased, the distribution of pore sizes shifted to larger pores (Figure 4f). The percentage of pores larger than 40 nm increased from 2.5% in the NC to 10.0, 13.7, and 24.5 in the 0.5, 1.5, and 3 hr OGD groups, respectively. Two final geometric features were calculated to better characterize nanoparticle behavior in the diseased brain microenvironment: trajectory boundedness (Supplemental Figure 1A) and efficiency (Supplemental Figure 1B). Boundedness reflects the proportion of a trajectory, which is restricted within a circular area, while efficiency reflects the ability of a nanoparticle to maximize displacement while minimizing distance traveled. 0.5 hr OGD caused a 1.1‐fold decrease in boundedness and 6.1‐fold increase in efficiency compared to that of NC. 1.5 hr OGD and 3 hr OGD also increased efficiency (by 6.4‐ and 5.4‐fold, respectively) but elicited no fold‐change on boundedness compared to that of NC.

### 3.5 Microglial uptake of nanoparticles is influenced by disease state and nanoparticle properties

**FIGURE 5:**
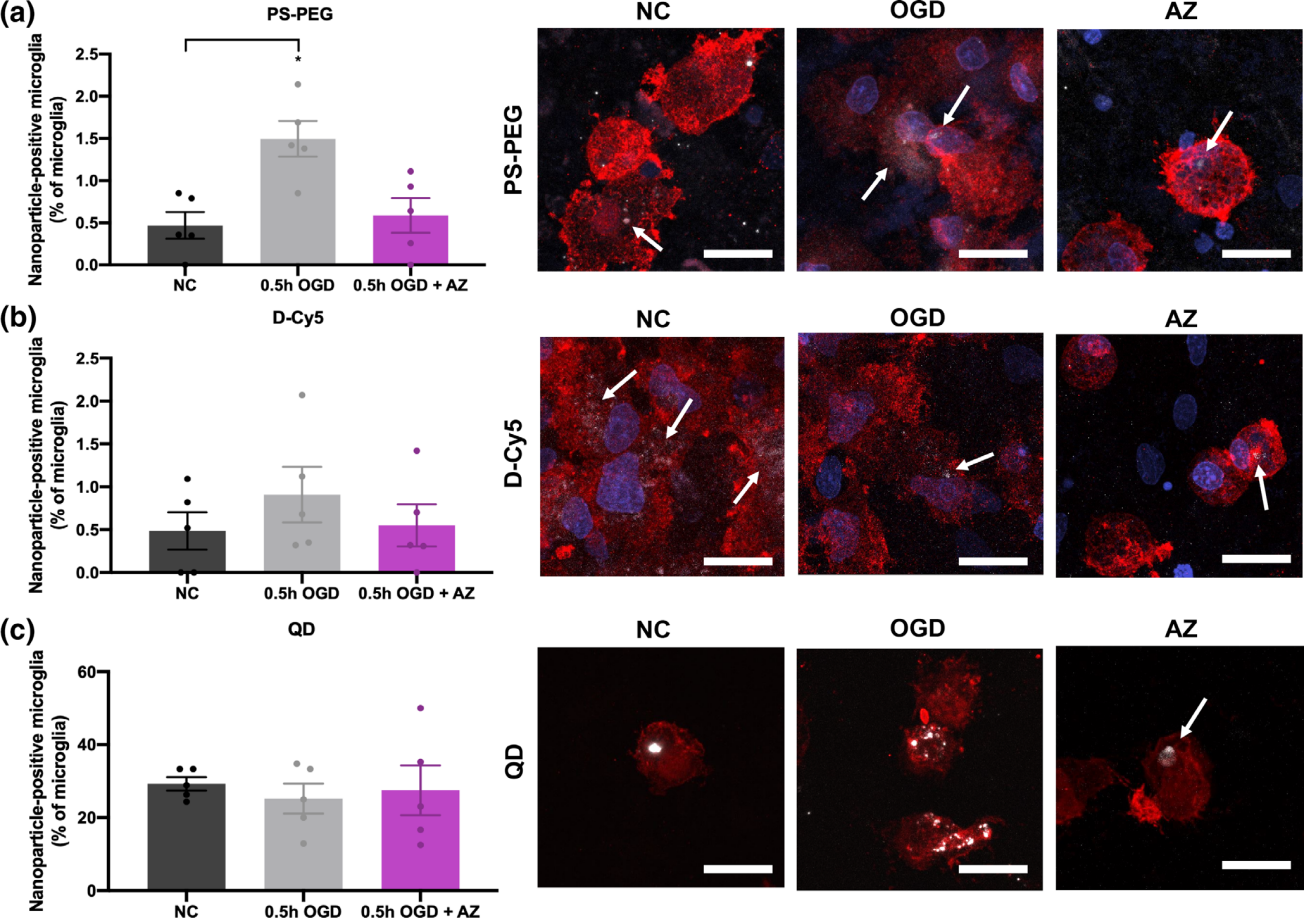
Microglial uptake of PS‐PEG, D‐Cy5, and QD nanoparticles at 5 hr for NC, 0.5 hr OGD, and 0.5 hr OGD + AZ treatment. Flow cytometry results of (a) PS‐PEG‐positive, (b) D‐Cy5‐positive, and (c) QD‐positive microglia as a proportion of all microglia in NC, 0.5 hr OGD, and 0.5 hr OGD + AZ groups. Colocalization of (a) PS‐PEG nanoparticles (white), (b) D‐Cy5 nanoparticles (white), and (c) QD nanoparticles (white) with NC, 0.5 hr OGD, and 0.5 hr OGD + AZ microglia (red) and cell nuclei (blue). All scale bars represent 20 μm. AZ, azithromycin; NC, normal control; OGD, oxygen–glucose deprivation; PS‐PEG, polystyrene‐poly(ethylene glycol); QD, quantum dots

We have confirmed OGD slice health can be recovered following AZ treatment and that particles can readily move within the OGD brain environment. We next sought to understand how injury and treatment influence the interaction of PS‐PEG, D‐Cy5, and QD nanoparticles with microglial cells. Using flow cytometry, we determined the proportion of microglia, which were nanoparticle‐positive in each treatment condition. We showed a significant increase in microglial uptake of PS‐PEG after OGD (PS‐PEG: 0.47% NC vs 0.5 hr OGD 1.50%, *p* = .016), and that AZ treatment reverted microglia to low‐uptake behavior (PS‐PEG: 0.59%) (Figure 5a). Application of 100x higher concentration PS‐PEG showed similar trends, with 27.3, 67.5, and 40.4% uptake in microglial cells for NC, 0.5 hr OGD, and 0.5 hr OGD + AZ conditions (Supplemental Figure 2). Microglial uptake of D‐Cy5 (Figure 5b) and QDs (Figure 5c) however, did not exhibit significant differences across all three conditions. QDs were internalized at much higher proportions than the other nanoparticle types across all experimental groups: median values for the 0.5 hr OGD group were 1.42, 0.68, and 25.0% for PS‐PEG, D‐Cy5, and QDs, respectively. Confocal images confirm that microglial uptake of nanoparticles occurred for all nanoparticle types and experimental conditions.

**FIGURE 6:**
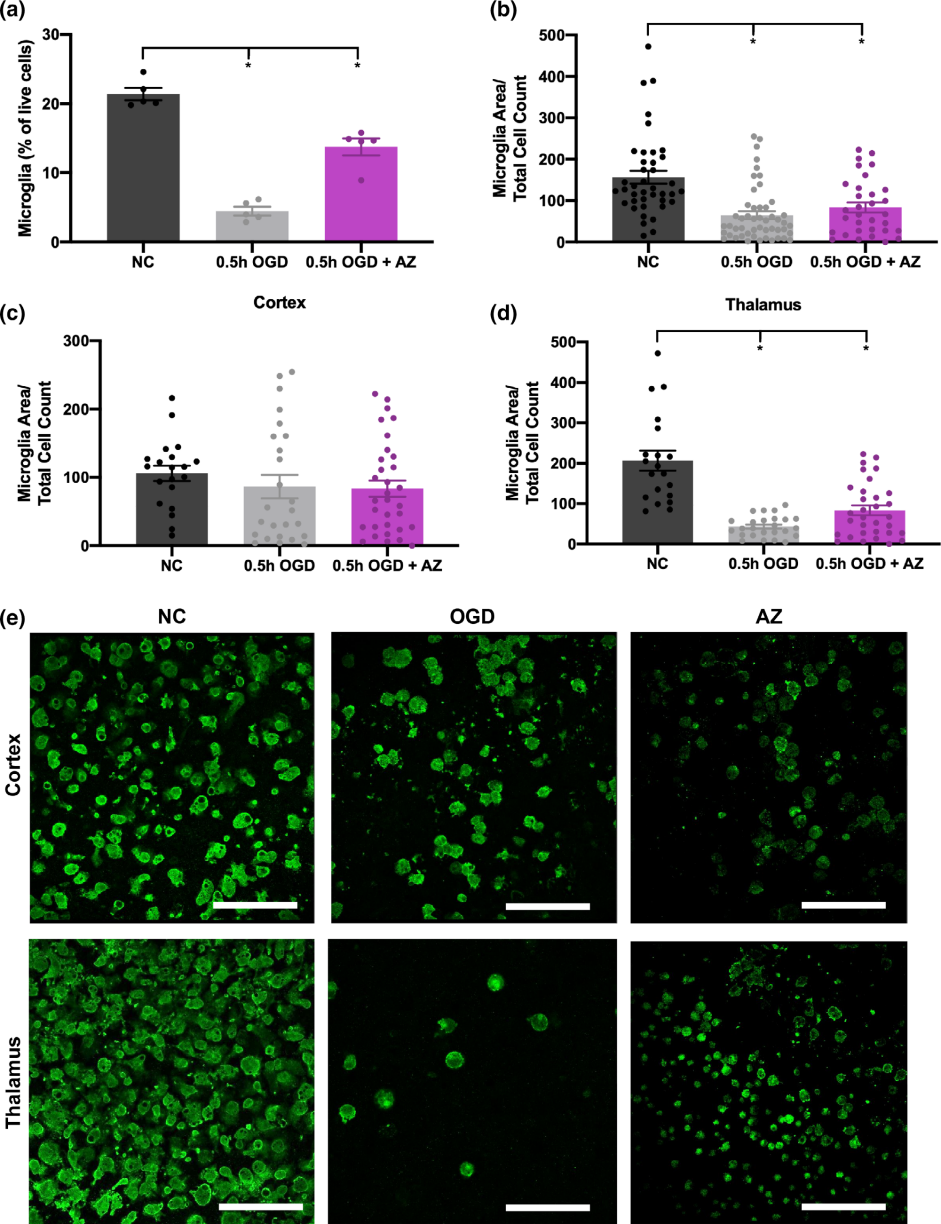
Microglial number decreases after OGD and is restored with AZ. (a) Flow cytometry results of microglia as a proportion of all live cells in the NC, 0.5 hr OGD, and 0.5 hr OGD + AZ groups 5 hr after injury. Microglial area of NC, 0.5 hr OGD, and 0.5 hr OGD + AZ slices normalized to DAPI‐positive cell count from images at 24 hr after injury, (b) for all images, (c) cortex alone, (d) thalamus alone and (e) Representative images of microglia (green) in the cortex and thalamus of NC, 0.5 hr OGD, and 0.5 hr OGD + AZ slices. All scale bars represent 100 μm. AZ, azithromycin; DAPI, diamidino‐2‐phenylindole; NC, normal control; OGD, oxygen–glucose deprivation

One important result from flow cytometry was the change in microglial number after injury and treatment. While microglia made up 20.33% of live cells in a NC slice, their proportion was significantly reduced after 0.5 hr OGD (3.99%), measured 5 hr after the end of injury (*p* = .016). However, treatment with AZ increased microglial proportion to 14.70% (*p* = .016 vs 0.5 h OGD) (Figure 6a). After 0.5 hr OGD, we saw a lower microglial percentage out of all live cells sustained for 25 hr (NC: 15.49%, 0.5 hr OGD: 4.00%, *p* = .024) as determined by flow cytometry (Supplemental Figure 3). This was further supported by quantitation of microglial area to total cell number at 24 hr after injury (Figure 6b). Interestingly, we observed a regional difference in microglial vulnerability. In the cortex, area covered by microglia did not change significantly across experimental condition (Figure 6c), but microglial area in the thalamus was significantly reduced after OGD (*p* < .001). Treatment with AZ increased microglial coverage (*p* = .031 compared to OGD) in the thalamus, although still to a reduced level from NC (*p* < .001) (Figure 6d). Representative confocal images from each region are shown in Figure 6e.

## 4 DISCUSSION

We investigated the nanoparticle‐ and disease‐dependent nature of nanoparticle‐microglia interactions using an OWH slice model of ischemic brain injury. Our first goal was to modulate ischemic brain injury severity by increasing OGD exposure times. There was an increase in cytotoxicity for all OGD exposure times compared to that of NC. Surprisingly, 1.5 and 3 h OGD exposure times resulted in lower cytotoxicity than 0.5 hr OGD exposure. Previous studies have demonstrated an increase in cytotoxicity from 0.5 to 1 hr of OGD ex vivo, and 60 min is regarded as the timeframe of OGD‐induced neuronal swelling followed by apoptotic and necrotic death.^45^, ^46^, ^47^, ^48^ In our study, exposure to 0.5 hr OGD was sufficient to induce significantly different outcomes than the NC condition, in agreement with other findings.^18^, ^49^, ^50^ Considering OGD mediates damage via oxidative stress, we measured the concentration of the redox buffering molecule GSH as another measure of slice health.^51^ All OGD exposure times reduced GSH concentrations, representing an oxidatively stressed environment.^52^

Microglia are potent cellular targets for drug delivery, due to their role propagating pathological processes after ischemic injury. Previous studies have demonstrated microglia‐specific drug delivery with PAMAM dendrimers, which was attributed to the increased phagocytic behavior of microglia in an activated state.^6^, ^12^, ^53^ Increased microglial uptake of dendrimers is also present after HI in mice in vivo, retinal HI injury in mice in vivo, and maternal inflammation‐induced cerebral palsy rabbits in vivo and ex vivo.^9^, ^54^, ^55^, ^56^ In all three models, microglia exhibited robust activation and proliferation, which was associated with an amoeboid cell shape, proinflammatory phenotype, and increased phagocytic behavior. However, in our study, dendrimer uptake was not significantly changed and no microglial proliferation occurred compared to that of the NC condition. In fact, microglial area from confocal images and microglial counts from FACS indicate a decrease in microglial number after OGD. One important note is the presence of a global inflammatory response in vivo that is absent from our slice preparation process ex vivo. Without systemic inflammatory contributions such as invasion by proinflammatory systemic immune cells, OGD alone may be insufficient to mediate microglial proliferation.^57^, ^58^, ^59^, ^60^ Thus, one major limitation of inducing injury ex vivo is the inability to fully represent complex pathological processes such as neuroinflammation that involve the immune system. Future studies may overcome this limitation by incorporating a strong inflammatory stimulus in vivo before brain extraction.

Microglial phenotypes in slice culture are also different than those represented in vivo^61^ and several studies have shown that nanoparticle uptake is correlated to microglial phenotype.^43^, ^62^ Accumulating evidence shows the importance of shape in characterizing microglial phenotype,^63^, ^64^ supporting microglial characterization using shape modes independently or in tandem with other classification systems, such as surface marker presentation, transcriptomics, or cytokine expression. Improving microglial characterization is essential to understanding microglial‐nanoparticle interactions, especially as the classical M1 and M2 microglial polarization phenotype schema progressively phases out.^65^ To better characterize the range of microglial morphologies in our OGD slice model, we used computer‐aided morphological analysis through application of the VAMPIRE software package. 0.5 hr OGD‐induced changes in microglial shape modes, which were reversed back to NC shape mode distributions upon AZ treatment. AZ has previously been shown to promote an anti‐inflammatory phenotypical change of macrophages and microglia.^21^ Although there remained some variation in frequency for the five shape modes for 0.5 hr OGD + AZ compared to that of NC, the ranking of shape mode frequencies from least (3) to greatest (2) followed the same ranking as the NC microglia shape mode frequencies. The VAMPIRE package enables in‐depth morphological analysis and detection of nuances in microglial shapes that encompass the heterogeneity of microglia that the human eye cannot detect. However, extracting intuitive shape characteristics of microglia from VAMPIRE is nontrivial given the complexity in interpreting the large dataset “machine–vision” classifications of the VAMPIRE package.^26^ Independent of shape mode, one readily interpretable morphological distinction between groups was that of microglial circularity, where high circularity corresponds to a nonbranching amoeboid morphology characteristic of a proinflammatory activated state.^64^ Although a value of one describes a perfect circle, some circularity values (1.2% of NC, 14.3% of 0.5 hr OGD, and 1.6% of 0.5 hr OGD + AZ) are slightly above 1 (1.12 max) due to errors in computer estimation of pixel perimeters.^66^ We found that 0.5 hr OGD increased microglial circularity and AZ prevented amoeboid morphology, reducing circularity levels to closer to that of NC microglia. Further work may better determine the association between nanoparticle uptake and microglial phenotype by analyzing additional cell features, such as degree of branching, branching polarization, and soma size, in combination with transcriptomic analysis, especially for nanoparticle containing cells.

In contrast to dendrimers, PS‐PEG nanoparticles exhibited increased microglial accumulation after OGD, and QDs were internalized at roughly equal proportions regardless of disease state. QDs were able to achieve orders of magnitude higher microglial accumulation after administration at the same dose as PS‐PEG and D‐Cy5. Our results indicate that microglial phagocytosis is highly dependent on nanoparticle platform, and disease‐induced changes in microglial behavior are not leveraged equally among all nanoparticle types. These platforms differ in size, rigidity, and chemical composition which can influence nanoparticle–cell interactions^67^, ^68^ suggesting that nanoparticle physicochemical parameters must be well‐tuned to achieve accumulation in target cells at sites of injury. For example, rigid lipid nanoparticles could more easily pass through cell membranes compared to less rigid nanoparticles.^69^ Previous work also supports microglial uptake of high‐rigidity nanoparticles, namely gold nanoparticles in vitro and silica and QD nanoparticles ex vivo and in vivo.^13^, ^70^, ^71^

Nanoparticle diffusive ability also plays a role in reaching target microglial cells and in achieving maximal therapeutic impact.^72^, ^73^ As evidenced by the MPT results after OGD, PS‐PEG nanoparticles exhibited more than 16‐fold higher diffusive ability compared to the NC condition. In this study, we directly confirmed diffusivity of PS‐PEG, but PEGylated QDs and PAMAM dendrimers have also been shown to move effectively within the brain parenchyma.^8^, ^11^, ^12^ After OGD, PS‐PEG nanoparticle trajectories were more efficient and the population of particles behaving subdiffusively decreased. This shift in diffusive transport modes may be associated with other disease‐mediated changes. For example, OGD‐induced cytotoxicity might reduce cell density and therefore decrease the likelihood of subdiffusive transport, which is characteristic of nanoparticles closely interacting with cellular compartments. Macroscopic ECS changes after ex vivo ischemic injury have also been previously reported, including an increase in striatal tortuosity and decrease in ECS volume fraction of brain tissue in the hippocampus and cortex.^74^, ^75^ The formation of dead‐space domains may explain both an increase in estimated ECM pore size and tortuosity.^76^ OGD also potentially increases the expression of ECM‐degrading matrix metalloproteinases (MMPs), as demonstrated in models of cerebral ischemia.^77^ The increase in ECM‐degrading enzymes can alter pore sizes and subsequently effect nanoparticle diffusion in the ECS. In this study, the distribution of ECS pore sizes did in fact shift to larger pores following OGD, which could be leveraged by the PEGylated nanoparticles for effective diffusive transport through injured tissue.

The proportion of pores greater than 40 nm increased with increasing exposure of OGD. Interestingly, an appreciable percentage of pores were predicted to be smaller than 40 nm, the particle diameter. Neutrally charged 40 nm PS‐PEG nanoparticles can evade many of the mechanisms by which extracellular movement is hindered,^8^ making diffusion predominantly influenced by steric interactions. While it is likely that some populations of particles are truly immobilized within pores, the Amsden obstruction model may be underestimating pore size by assuming nanoparticles are completely inert. PEGylated nanoparticles may interact with microglia, other cell types, and various components of the ECM, which hinders transport and may skew pore size estimates. Although the average pore size was smaller than previously calculated, the range of pore sizes identified in this study is similar to previous findings using MPT analysis in brain slices.^78^ The use of 40 nm particles for prediction, compared to 40, 100, and 200 nm particles used by Nance et al,^8^ could underestimate the true size of pores >40 nm and skew the calculated average pore size to be smaller. Regardless, the model was applied equally across all experimental groups and therefore provides insight into the differences existing between conditions in this study. Nanoparticle probe size, different culturing conditions, and DIV can each alter MPT analysis and the application of the Amsden obstruction model, requiring further investigation in the OGD model.

One benefit of the OWH slice platform for furthering this work is the ability to study regional variability in response to injury. After both 1.5 and 3 hr OGD, nanoparticles in the striatum had consistently faster *D*_eff_ compared to those in the cortex. Microglial area coverage also indicated a greater injury response in the midbrain. Only the thalamus, not the cortex, showed a decrease in microglial area coverage after 0.5 hr OGD. Although we did not probe for a mechanism to explain regional differences, such an investigation can have important implications for therapeutic development. One recent study investigated the injury‐resistant nature of the hypothalamus region and identified that slow neuronal depolarization in the region may be one native mechanism of neuroprotection.^79^ Enhanced therapeutic penetration within diseased brain regions could reduce requisite dose amount and frequency and avoid inadvertent cytotoxicity on healthy tissue. Due to the intricate balance of pro‐ and anti‐inflammatory activity in the brain microenvironment after disease, region specific control can also reduce over‐scavenging of ROS or excessive inhibition of inflammatory processes that could interrupt healthy cellular function or exacerbate damage.^80^, ^81^, ^82^ Given that ischemic injury manifests in regional patterns in multiple phases,^83^, ^84^ the continued investigation of regional variations in the brain could inform therapeutic strategies that are highly advantageous in combating immediate and ongoing regionally‐dependent disease sequelae.

Further cross‐platform investigation will be important to elucidate advantageous nanoparticle characteristics, in addition to diffusive ability, size, rigidity, and surface functionalization, for microglial‐targeted drug delivery after injury. Microglial uptake of additional nanoparticle platforms, such as nanocrystals or polymeric micelles, has been understudied yet may elucidate new therapeutic avenues. Importantly, immediate AZ treatment of OGD‐injured slices returned microglial uptake behavior of all nanoparticle types closer to that of NC conditions, suggesting that modulated microglia of recovered brain tissue behave similarly to healthy microglia. While this work primarily studied AZ modulation of slice health and microglial behavior, we also demonstrated that application of SOD or AZ is neuroprotective after ischemic injury. Brain slices treated with either therapeutic had significantly reduced cell death over 24 hr, and AZ additionally prevented a decrease in GSH concentration, indicative of an inhibition of oxidative stress and cell damage. SOD efficacy is well defined by scavenging of superoxide anion, which plays a damaging role in excitotoxicity, but the exact mechanism of AZ therapeutic efficacy remains to be elucidated in the brain.^85^, ^86^ Regardless of therapeutic mechanism, therapeutic reversion of OGD‐induced changes offers promising implications for drug delivery strategies. If SOD or AZ were delivered via rigid carriers similar to PS‐PEG nanoparticles that exhibited increased diffusion and microglial uptake after OGD exposure, drug distribution would favorably accumulate in diseased regions. After therapeutic release and reduction of disease phenotype, subsequently administered nanoparticle doses would preferentially sequester in ongoing injury sites compared to recovering or healthy tissue environments. Further investigation of strategies to modulate nanoparticle diffusivity and microglial uptake may result in more effective methods of nanoparticle‐mediated therapeutic delivery.

## 5 CONCLUSION

In this work, we probed the effect of OGD‐induced brain injury and AZ treatment on nanoparticle interactions with microglia. First, we determined the effect of OGD exposure on markers of injury severity, including cell death and oxidative stress. We observed significant injury responses after 0.5 hr OGD exposure: a 54.3% increase in cytotoxicity, a 1.7‐fold decrease in GSH concentration, and a larger pore distribution in the ECS. We observed an OGD‐induced shift in microglial morphology toward more heterogeneity in shapes with overall increased circularity and a decrease in microglial density. Nanoparticle interactions with microglia were dependent on both the nanoparticle platform as well as treatment condition. After 0.5 hr OGD, microglial internalization of PS‐PEG was increased, but uptake of QDs or dendrimers was not enhanced, indicating an important role of nanoparticle material identity in determining extent of phagocytosis after injury. OGD injury did not impede nanoparticle mobility; PS‐PEG nanoparticles had a 16.7‐fold increase in diffusion after 0.5 hr OGD compared to that of NC. Treatment with AZ not only effectively reduced OGD cytotoxicity and GSH depletion, but also reverted nanoparticle uptake behavior of PS‐PEG and microglial morphology toward that of NC. This study shows OWH slices enabled region‐dependent nanoscale probing of live tissue to identify cellular and microenvironmental changes in diseased and recovering brain that can be leveraged for cell‐specific uptake of nanoparticles. In addition, we demonstrate that in ischemic conditions, nanoparticle fate is platform‐dependent, providing insights into therapeutic strategy for targeting microglial cells to combat neurological disease.

## CONFLICT OF INTEREST

The authors declare no potential conflicts of interest.

## Supporting information


**Appendix** **S1**: Supporting Information.
