# Supplementary material for: Nanoparticle‐microglial interaction in the ischemic brain is modulated by injury duration and treatment
Source: Bioeng Transl Med. 2020 Aug 15;5(3):e10175. doi: 10.1002/btm2.10175 (PMC7510458; doi:10.1002/btm2.10175)
Supplement: Supplementary file 1 — Appendix S1: Supporting Information. [file BTM2-5-e10175-s001.docx]

**Supplemental Information**

Nanoparticle-microglial interaction in the ischemic brain is modulated by injury duration and treatment

Andrea Joseph^1#^, Rick Liao^1#^, Mengying Zhang^2^, Hawley Helmbrecht^1^, Michael McKenna^1^, Jeremy Filteau^1^, and Elizabeth Nance^1-4 *^

^1^Department of Chemical Engineering, University of Washington, Seattle, Washington, 98195

^2^Molecular Engineering and Sciences Institute, University of Washington, Seattle, Washington, 98195

^3^Department of Radiology, University of Washington, Seattle, Washington, 98195

^4^eScience Institute, University of Washington, Seattle, Washington, 98195

#Authors contributed equally to this work.

*Corresponding author: eanance@uw.edu

**Supplemental Figures**

**
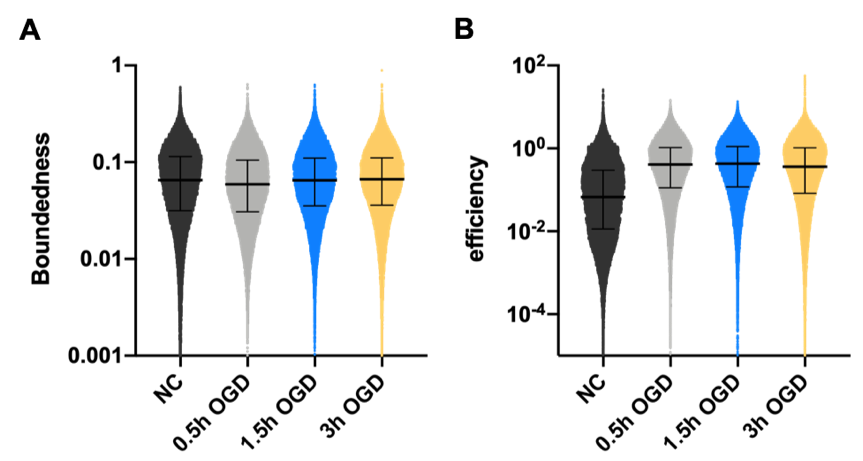
**

**Supplemental Figure 1.** Nanoparticle boundedness (A) and efficiency (B) calculated for each trajectory from all videos (n=10) in all slices (n=3) per group (1 dot = 1 trajectory).

**Supplemental Figure 2.** Microglial uptake of 100ng/μL PS-PEG nanoparticles. A) Representative confocal images of PS-PEG (white) and microglia (red) after administration of 100ng/μL(a 100-fold higher dose than the representative data in Figure 5). All scale bars represent 20 µm and cell nuclei (blue) are also shown. B) Flow cytometry results indicate nanoparticle-positive microglia as a proportion of all microglia in each treatment group.

**Supplemental Figure 3.** Flow cytometry results of microglia as a proportion of all live cells in the NC and 0.5h OGD groups at 25h after injury

**Supplemental Tables**

**Supplemental Table 1.** Average circularity of microglia by shape mode for a given experimental condition.

| **Experimental Condition** | **Shape Mode** | | | | | |
| --- | --- | --- | --- | --- | --- | --- |
|  | **1** | **2** | **3** | **4** | **5** | **Average** |
| **NT** | 0.53 | 0.48 | 0.50 | 0.53 | 0.47 | 0.50 |
| **OGD** | 0.63 | 0.59 | 0.78 | 0.85 | 0.61 | 0.69 |
| **OGD+AZ** | 0.55 | 0.48 | 0.57 | 0.57 | 0.49 | 0.53 |

**Supplemental Table 2.** Physicochemical properties of PS-PEG used in MPT and nanoparticle uptake studies. A non-PEG coated PS particle is included for comparison. Nanoparticles (n=3 batches per particle type) were characterized in terms of hydrodynamic diameter, mean surface charge (ζ-potential), and the polydispersity index (PDI) by dynamic light scattering at 25°C and pH 7.2 in 10 mM NaCl.

| **Nanoparticle Type** | **Number Mean**  **± SEM (nm)** | **Intensity Mean**  **± SEM (nm)** | **PDI** | **ζ-potential ± SEM (mV)** |
| --- | --- | --- | --- | --- |
| **PS-COOH** | 39.1 ± 0.49 | 53.2 ± 0.58 | 0.044 | -37.5 ± 0.74 |
| **PS-PEG** | 51.4 ± 0.96 | 66.9 ± 0.40 | 0.027 | -0.59 ± 0.10 |

**Supplemental Methods**

*Animals*

Time-mated pregnant female Sprague–Dawley (SD) rats (virus antibody-free CD® (SD) IGS, Charles River Laboratories) were purchased and arrived on estrous (E) day 17. Dams were housed individually and allowed to acclimate to their environment for a minimum of 3 days prior to delivering. The day of birth was defined as postnatal (P) day 0. Litters containing both sexes were cross-fostered and culled to 12 animals early after birth. Before and after the experiment, each dam and her pups were housed under standard conditions with an automatic 12h light/dark cycle, temperature range of 20–26°C, and access to standard chow and autoclaved tap water *ad libitum*. The pups were checked for health daily.

*Brain slice preparation and culturing medias*

We use OWH slices to investigate nanoparticle-cell interactions. OWH slices have benefits over both *in vitro* and *in vivo* approaches. *In vitro* cell culture inadequately captures the full complexity of the local barriers and brain microenvironment that represents HI pathogenesis. On the other hand, *in vivo* studies are costly, especially when incorporating all engineering and therapeutic controls, and have high biological variability that confounds preclinical results. To prepare OWH slices, on P7 healthy male SD rats were injected with 100μL pentobarbital, followed by rapid decapitation with surgical scissors once the body was non-responsive. After removing the brain, under sterile conditions, the brain was split into hemispheres with a sterile razor blade and sliced into 300μm sections with a McIlwain tissue chopper (Ted Pella). Brain slices separated in dissecting media were transferred onto 35-mm 0.4-μm-pore-sized membrane inserts (Millipore), and placed within a 6-well plate (CytoOne) containing 1 mL slice culture media (SCM). Brain slice dissecting media consisted of 0.64% w/v glucose, 100% HBSS (Hank’s Balanced Salt Solution), and 1% penicillin. SCM was made of 5% horse serum (ThermoFisher) 50% MEM (minimum essential media), 45% HBSS, 1% GlutaMAX, and 1% penicillin). All media added to the wells was pre-warmed at 37°C. MEM was purchased from Life Technologies, glucose from Sigma, and HBSS, GlutaMAX, and penicillin from Gibco. OGD media consisted of 150 mM sodium chloride (NaCl, Sigma), 2.8mM potassium chloride (KCl, Sigma), 1mM calcium chloride (CaCl_2_, Sigma), and 10mM 4-(2-hydroxyethyl)piperazine-1-ethanesulfonic acid buffer solution (HEPES, Gibco) in deionized water titrated to pH 7.4 with 1M hydrochloric acid (ThermoFisher) or 1M sodium hydroxide (ThermoFisher), and bubbled with nitrogen gas (Praxair) for 10 minutes. All media added to the wells was pre-warmed at 37°C. MEM was purchased from Life Technologies, glucose from Sigma, and HBSS, GlutaMAX, and penicillin from Gibco. The live slices incubated in a CO_2_ incubator (ThermoFisher) at 37°C with constant humidity, 95% air, and 5% CO_2_ to equilibrate after the mechanical stress of slicing before continuing experiments. For samples undergoing OGD, supernatant was removed, and the 6-well plate well was rinsed once with 1mL OGD media. After removal of the rinse media and addition of 1mL OGD media to the well, membrane inserts were placed back in the well. The 6-well plates with OGD samples were placed in a Hypoxia Incubator Chamber (STEMCELL Technologies) and placed in 37°C incubator. The chamber was flushed with nitrogen gas (Praxair) for 10 min, followed by clamping of the tubing. The OGD slices continued incubating for the remainder of the 0.5h, 1.5h, or 3h. After OGD, supernatant was removed, and the 6-well plate wells were rinsed once before adding 1mL fresh SCM and returning membrane inserts into the well. Sample conditions included NC, 0.5h, 1.5h, or 3h OGD, 0.5h OGD+AZ, and 3h OGD+SOD.

*Glutathione (GSH) assay*

At t=24h for all sample conditions, brain slices were frozen at -80°C for GSH detection with the GSH/GSSG ratio detection assay kit fluorometric green (Abcam). Three brain slices approximating 15g of brain tissue were processed for each sample. Samples were processed following the manufacturer’s instructions and analyzed for fluorescence at 490/520 excitation/emission on a Cytation 3 UV-Vis Spectrophotometer (BioTek Instruments) to measure the extent of thiol green indicator reaction with GSH. Adjusting for volume resuspension and dilutions, GSH concentrations were reported as μmoles GSH per gram of initial brain tissue.

*Propidium iodide (PI) and microglia staining*

At t=24h for all sample conditions, slices were stained with 1mL SCM with 5μg PI in SCM for 45 min in the CO_2_ incubator. Slices were washed twice for 5 min with SCM, followed by a 1h wash with SCM in the CO_2_ incubator, and finally fixation with 10% phosphate buffered formalin (Fisher Scientific) for 1h at room temperature. Fixed slices were stained with rabbit anti-Iba1 (Wako 019-19741, 1:200) for 6h followed by goat anti-rabbit AlexaFluor 488 (Life Technologies A11034, 1:500) for 2h and 1:10,000 4′,6-diamidino-2-phenylindole (DAPI, Invitrogen) for 15 min with two washes between each step at room temperature, and stored at 4°C with 1mL 1x PBS for temporary storage before imaging.

*Nanoparticle characterization and multiple particle tracking (MPT) in OWH brain slices ex vivo*

Nanoparticles were characterized by dynamic light scattering (DLS) to measure hydrodynamic diameter and polydispersity index (PDI) and by laser Doppler anemometry to measure ζ-potential (Supplemental Table 1). Both DLS and laser Doppler anemometry were carried out using a Zetasizer Nano ZS (Malvern Panalytical). At t=24h for NC, 0.5h, 1.5h, and 3h OGD samples, SCM was exchanged for a nuclear staining solution consisting of 1mL SCM and 5 drops NucBlue Live ReadyProbes Reagent (Hoechst 33342, ThermoFisher). Slices were stained for 1h then washed twice with 1mL warm SCM for 5 min each. Following the second wash, 0.5µL injections of 40nm PS-PEG nanoparticle stock diluted in 1x PBS to a concentration of ~0.0011% (w/w) solids were carried out in each slice using a 10µL glass syringe (model 701, cemented needle, 26-gauge, Hamilton Company). Four injections were made in the cortex and three in the striatum. Slices were transferred to a temperature-controlled imaging incubation chamber maintained at 37°C, 5% CO_2_, and 80% humidity, where they remained throughout the imaging window. Video acquisition began 30 min after nanoparticle injection and was completed within 2h. A total of five videos were collected from the cortex and striatum of each slice. Videos were collected at 30.3 frames-per-second and 100x magnification (0.07µm/pixel) for 651 frames via fluorescent microscopy using a cMOS camera (Hamamatsu Photonics) mounted on a confocal microscope.

Nanoparticle trajectories, trajectory mean squared displacement (MSD), effective diffusion coefficients (D_eff_), and features were calculated via diff_classifier, a Python package developed within our group. The diff_classifier package is publicly available on GitHub at Nance-Lab/diff_classifier and is published in the *Journal of Open Source Software*.^30^ To obtain distributions of brain extracellular matrix (ECM) pore sizes, the measured D_eff_ were fit using an obstruction-scaling model described by Amsden,^32^

|  | $\frac{D_{eff}}{D_{0}}=exp\left[ -\pi\left( \frac{r_{s}+r_{f}}{\xi+2r_{f}} \right)^{2} \right]$ | (1) |
| --- | --- | --- |

where $D_{eff}$ and $D_{0}$ are diffusion coefficients within ECM and in a free medium, respectively, $r_{s}$ is the critical limiting radius (radius of the nanoparticle probe in this instance), $r_{f}$ is the radius of the polymer chains, and $\xi$ is the average mesh size of the network. For the purposes of this study, $D_{0}$ was calculated as the theoretical diffusion coefficient of nanoparticles in water at 20°C using the Stokes-Einstein equation for spherical particles. $r_{f}$ was assumed to be equivalent to the thickness of hyaluronic acid, a major component of brain ECM. For the purposes of this study, we assumed brain ECM consisted solely of hyaluronic acid fibers whose thickness has been determined previously from atomic force microscopy.^34^ The remaining variables, $r_{s}$ and $D_{eff}$, were determined experimentally. The model has demonstrated accuracy when applied to both homogenous^32^ and heterogeneous^31^ hydrogels, despite assuming straight, randomly oriented polymer fibers, a property characteristic of heterogeneous hydrogels. The model relies on the following assumptions:

1. The nanoparticles are hard spheres.
2. The intermolecular forces of attraction between nanoparticles and polymer chains are negligible.
3. The polymer chains act only as steric obstacles to diffusion.
4. The polymer chains are immobile relative to the mobility of the nanoparticles over the time scale of the diffusion process.
5. The distribution of pores between polymer chains can be approximated by a random distribution of straight fibers, described by the Ogston expression.^33^

The intensity-mean hydrodynamic radius of the 40nm PS-PEG nanoparticles, as determined by DLS, was used as the critical limiting radius.

*Immunofluorescence for nanoparticle co-localization in microglia and neurons*

To probe nanoparticle interactions with microglia, at t=1h PS-PEG and D-Cy5 (10µL of 1 ng/µL) were topically pipetted on NC, 0.5h OGD, and 0.5h OGD+AZ OWH slices. The slices were then incubated at 37°C and 5% CO_2_ for 4h to allow the nanoparticles to diffuse through the brain tissue and interact with microglia. At t=5h, the slices were washed with 1x PBS to remove excess nanoparticles that were not taken up intracellularly. After fixation, the slices were washed twice and stained with primary antibodies for neurons (mouse anti-MAP2, Abcam ab11268, 1:200) and microglia (rabbit anti-Iba1, Wako 019-19741, 1:200) for 6h. Secondary antibodies donkey anti-mouse Alexa Fluor 488 (Life Technologies A21202, 1:500) and donkey anti-rabbit Alexa Fluor 546 (Life Technologies A10040, 1:500) were applied to slices for 2h, followed by DAPI staining (1:10,000). Slices were washed twice between each staining step. Z-stack images of nanoparticles and cells were performed at 4x zoom at 60x magnification using a Nikon confocal microscope with a z-step of 0.1-0.2µm. All the instrument and camera settings among samples were kept the same.

*Flow cytometry with OWH slices*

Flow cytometry was performed on NC, 0.5h OGD, and 0.5h OGD+AZ OWH slices. Each group included n=5 samples per condition and 3 slices per sample. At t=1h, nanoparticles were topically pipetted on the OWH slices (10μL of 1ng/µL PS-PEG, D-Cy5, QD, or PS-COOH). Slices for each condition were placed in 1mL Accutase (Millipore Sigma) at t=5h. Samples were homogenized and filtered with a 250µm Pierce Tissue Strainer (ThermoFisher) to remove large debris. Following a wash step, microglia were isolated using a Percoll (Sigma-Aldrich) gradient. The cells were then stained with DAPI (1:10,000) and FITC anti-rat CD11b antibody (BioLegend, 1:200). Appropriate controls for CD11b gating were done with an aliquot of the control sample. The BD LSRII (BD Biosciences) machine recorded cells in each sample with fluorescence in the DAPI, CD11b, and Cy5 (for PS-PEG and D-Cy5) or BV650 (for QDs) channels until 100,000 events (live cells) were reached. Analysis of the cytometry data was performed in FCS Express 7 Research by thresholding the far-red events appropriately compared to a no-particle control. For microglial number data, slices with PS-COOH were processed at t=25h.
